# Loss Limits as a Predictor of Future Gambling Behavior

**DOI:** 10.1007/s10899-025-10446-3

**Published:** 2025-11-18

**Authors:** Elise Victoria Tørdal, Tony Leino, Ståle Pallesen

**Affiliations:** 1 Department of Psychosocial Science, University of Bergen, Bergen, P.O. Box 7807, N-5020 Norway; 2 Norwegian Competence Center for Gambling and Gaming Research, University of Bergen, Bergen, P.O. Box 7807, N-5020 Norway

**Keywords:** Problem gambling, Responsible gambling, Pre-commitment, Loss limits, Long-term predictive effects, Harm reduction

## Abstract

In 2021, the Norwegian government implemented a policy requiring individuals gambling with the horse betting monopolist, Norsk Rikstoto, to set personal loss limits, capping the maximum loss limit at 20,000 NOK (10 NOK ∼ 1 €) per month. The present study investigated whether loss limits set in 2021 could predict gambling behavior in 2023, and whether loss limits were associated with the proportion of moderate-to-high risk gamblers. *Method*: The dataset encompassed the entire population of customers who participated in horse betting in 2021 and at least once in 2023, totaling 104,413 individuals (31.6% women). It included data on expenditure, net winnings, and the number of bets placed, as well as each customer’s classification within a problem gambling risk category for 2023. While no clear association was found between 2021 loss limits and 2023 gambling expenditure, higher loss limits predicted more bets, lower net winnings, continued gambling, and a greater likelihood of being classified as moderate-to-high risk gamblers by the operator’s monitoring system. *Conclusion*: The findings show that loss limits may be a valuable predictor of future gambling behavior at the group level.

**Supplementary Information:**

The online version contains supplementary material available at 10.1007/s10899-025-10446-3.

Problem gambling (PrG) entails compulsive and persistent engagement in gambling behavior despite adverse consequences, resulting in significant detriments in terms of finances, relationships, emotions, health, culture, performance, and/or legal functioning (Bolen & Boyd, 1968; Langham et al., 2016). A meta-analysis from 2024 reported that 46.2% of the global adult population had engaged in gambling within the past 12 months. Of these, 8.7% were classified as engaging in risk gambling, and 1.4% met criteria for problematic gambling (Tran et al., 2024). In Norway, the most recent gambling prevalence study from 2022, showed that 7.8% of the population were classified as low-risk gamblers, 2.3% as moderate-risk gamblers, and 0.6% as problem gamblers (Pallesen et al., 2023). A total of 2.1% of the Norwegian population reported gambling-related harm of at least some impact, with finances (6.1%), well-being (5.5%), and health-related (3.3%) harms being the most frequent. Compared to the previous population survey conducted in 2019 (Pallesen et al., 2020), the 2022 study indicated a reduction in the prevalence of gambling problems (Pallesen et al., 2023). Still, a noteworthy proportion of the Norwegian population struggles with gambling problems, and for each problem gambler, an estimated five to 15 others are adversely affected (Kalischuk et al., 2006). This reflects a need for regulatory measures and research to better understand and mitigate the problems and harms caused by gambling.

A substantial body of research highlights notable gender differences in gambling behaviors and the prevalence of PrG. For example, men are more likely than women to engage in gambling activities and consistently report higher rates of PrG (Grant & Kim, 2002; Wenzel & Dahl, 2009). However, despite higher overall prevalence of gambling problems among men, women, consistent with the telescoping hypothesis, tend to develop gambling problems later in life but have a faster progress towards problems than men (Syvertsen et al., 2023a). This underscores the need to investigate gambling behavior through a gender-sensitive lens.

In efforts to reduce overall harm associated with gambling, the concept of responsible gambling (RG) tools has been introduced. Blaszczynski et al. (2004) describe RG tools as policies and practices designed to prevent and mitigate potential the potential negative consequences of gambling. Pre-commitment tools, for example loss limits, enable players to determine in advance the amount of money they intend to allocate to gambling within a specified timeframe. These limits may be optional – allowing players to choose whenever to use them – or mandatory, requiring players to set limits in order to access the services of a particular gambling operator (Wood & Griffiths, 2010).

The regulation of gambling activities, particularly through mechanisms such as loss limits, has been a focal point of policy interventions aimed at mitigating gambling-related risks (Auer & Griffiths, 2013). A study conducted in Nova Scotia, Canada, found that implementing monetary limits generally led to a reduction in total gambling expenditure (Focal Research Consultants, 2007). Similarly, a study involving 5,000 registered players in Austria who opted to impose limits on their play with deposit restrictions permitted up to a maximum of €800 per week found that limit-setting effectively targeted and influenced the behavior of the most intensive players, leading to a reduction in their spending. The analysis also indicated that, in most cases, monetary limits had a greater impact on gambling behavior than restrictions on the duration of play (Auer & Griffiths, 2013). However, when loss limits are voluntary, studies indicate that their up-take and effects are limited (Delfabbro & King, 2021; Marionneau et al., 2025).

In Norway, gambling is offered through a state-controlled monopoly system managed by Norsk Tipping and Norsk Rikstoto (Rossow & Hansen, 2016). With the exception of bingo and certain lotteries regulated separately, Norsk Tipping offers lotteries, sports betting, VLTs, and online casino games, while Norsk Rikstoto holds exclusive rights to horse race betting. Regarding RG tools and particularly pre-committed loss limits, the regulatory framework in Norway has undergone significant development in recent years. In 2016, Norsk Tipping became the first gambling operator globally to implement a mandatory maximum loss limit across all games, requiring players to set a personal limit capped at 20,000 NOK per month (10 NOK ~ 1€) (Norsk Tipping, 2023). In 2021, the Norwegian government extended this requirement to Norsk Rikstoto (Rikstoto, 2020). This regulatory shift presents a unique opportunity to examine not just whether loss limits affect behavior, but whether the amount set under this mandatory system predicts future gambling outcomes.

While previous studies have examined whether setting a loss limit reduces gambling behavior (Auer et al., 2020), there is little/no evidence on whether the size of loss limits in general has long-term predictive value. A high limit may offer minimal constraint and reflect heavier gambling involvement, whereas a low limit may indicate a more cautious or conservative gambling style. As most existing research focuses on outcomes within days or weeks, this study examines whether loss limits set in 2021 predict gambling behavior and risk two years later, providing a rare long-term perspective on responsible gambling outcomes.

Against this backdrop, the present study aimed to investigate whether loss limit settings among Rikstoto customers in 2021 could predict gambling behavior in 2023. Gambling behavior was operationalized as total expenditure, net winnings, number of bets placed, and classification within PrG risk categories in 2023. To the best of our knowledge, no prior study has specifically examined the long-term relationship between mandatory loss limits on subsequent gambling behavior. The findings aim to contribute to our understanding of responsible gambling practices and inform potential strategies for minimizing risks associated with mandatory loss limits in a gambling context.

## Method

### Samples and Procedures

Norsk Rikstoto (NR) provided gambling behavior data encompassing the entire population of customers who participated in gambling activities at least once between January 2021 and December 2023. The raw dataset consisted of weekly aggregated records, including variables related to gambling behavior (expenditure, net winnings, and the number of bets placed) as well as gambling risk indicators derived from an algorithm designed to assess gambling-related problems. The dataset included a total of 7,395,336 observations (24.9% women) nested within 277,517 customers (37.2% women). A total of 14,773 observations within 13,549 customers (*M*_age_=61.3 years, 37.7% women) with 0 gambling expenditure were removed from the dataset. Additionally, 10 observations within 9 customers (*M*_age_=65.0 years, 11.1% women) who placed 0 bets across all weeks were also removed. Taking these exclusions into account, the analytic sample consisted of 7,380,553 observations (24.9% women) within 277,053 individuals (37.1% women). To examine the relationship between loss limits in 2021 and gambling behavior in 2023, only data from customers who gambled at least once in both 2021 and 2023 were included in the analysis.

### Variables

#### Gambling Behavior

Gambling behavior (expenditure, number of bets placed, and net winnings) was aggregated across 2023, providing a two-year follow-up from the loss limits set in 2021. Using data from the full calendar year reduced the impact of short-term variability (e.g., temporary spikes, seasonal fluctuations, and pauses) and was intended to capture longer-term behavioral patterns, consistent with the study’s focus on the predictive value of loss limits over time. Net winnings were calculated as the difference between the income from winnings and the expenditure.

## Loss Limits

Loss limits were defined as the size of the last loss limit set by each customer in 2021, to reflect the most recent and likely most deliberate limit-setting decision before the follow-up period. Monthly loss limits were categorized into six categories: 0) No loss-limit (a default limit of 5,000 NOK was automatically set by Norsk Rikstoto in the absence of user selection [Norsk Rikstoto, 2020]), (1) 0.5–5.5,000 NOK, (2) 5,001–12,000 NOK, (3) 12,001–20,000 NOK, (4) 20,001–50,000 NOK, and (5) 50,001 NOK or more. The intervals were designed to be broad enough to capture meaningful differences between customer groups, while still offering sufficient granularity for detailed analysis. Further, categorization was used as the relationship between loss limits and gambling behavior is not linear and the categorizes used reflect commonly set loss limits.

**Fig. 1:**
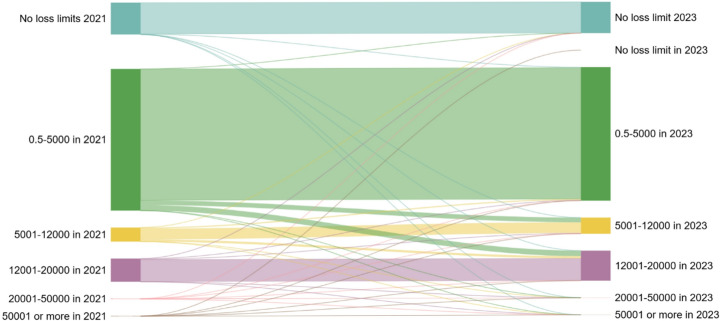
Sankey plot showing the correspondence between the last loss limit set in 2021 and the last loss limit set in 2023

While loss limits are generally capped at 20,000 NOK, some players are recorded with limits exceeding this threshold. This resulted from a rollover system, which transfers any unused portion of the loss limit to the next 30-day period, with rollovers permitted for up to three months. Consequently, players who did not use their full limit could accumulate a higher effective limit over time. Additionally, winnings from January 1, 2021, that players opted to reinvest were included in a rolling 365-day period. Positive winnings carried over from 2020 were also considered, potentially increasing a player’s available limit (Rikstoto, 2020). Notably, the reinvestment feature is optional and must be actively enabled by the player. For most customers the last loss limit in 2021 corresponded to their last loss limit set in 2023 (see Fig. 1).

### Risk of Gambling Problems

The data included information on each customer’s risk of gambling problems, as classified by Mentor, a machine learning–based algorithm that continuously evaluates customer behavior and classifies them into four groups: (1) No-risk, (2) Low-risk, (3) Moderate-risk, and (4) High-risk. Risk classification was based on trends in variables such as number of days played, number of bets, amounts lost and loss limits (H. Skappel, personal communication, October 15, 2024). Because changes in loss limits are included in this classification, it is not fully independent of the predictor variable in this study and should thus be interpreted accordingly. Classifications are updated daily, allowing customers to move between multiple risk categories within a single month (Rikstoto, 2022).

The risk of gambling problems used in the analyses was operationalized as each customer’s highest observed risk level in 2023. This variable was then dichotomized into two categories: 0) *No-to-low risk gamblers* (comprising categories 1 and 2), and 1) *Moderate-to-high risk gamblers* (comprising categories 3 and 4). This grouping was made in order to differentiate between customers with no or small risk and those with a clearly elevated risk of developing gambling problems, hence creating groups with meaningful distinctions.

## Statistical Analyses

For all analyses, the main exploratory variable was the size of loss limit in 2021, stratified by sex and adjusted for customer age in 2021. Linear regression models were used for continuous outcomes (i.e., expenditure and net winnings in 2023). A zero-truncated Poisson model was applied to count outcomes (i.e., number of bets placed in 2023), and binary logistic regression model was used to estimate the odds of being classified as a moderate-to-high risk gambler versus a no-to-low risk gambler in 2023. All analyzes were conducted using Stata version 18.0 (StataCorp LLC, 2023).

Statistical significance testing (i.e., null hypothesis testing) was not conducted between groups, as the analysis included the entire population. Since no sampling was involved, hypothesis testing was deemed unnecessary (McNulty, 2022). The analytical scripts used in this study are provided in the Appendix.

### Ethics

The study was, according to the Norwegian Health Research Act, exempted from ethical approval as all data were provided anonymously. No specific funding was provided for the study.

None of the authors have any conflicts of interest that could bias this work. Informed consent was not provided as all analyses were based on played account data that the provider, Rikstoto, can make available for research purposes.

## Results

### Description of Population

A total of 104,413 customers (33.6% women) gambled with Norsk Rikstoto at least once in both 2021 and 2023. The mean age was 56.9 years (*SD* = 14.2). Although men (*M* = 57.2 years, *SD* = 14.2) were slightly older than women (*M* = 56.3 years, *SD* = 14.3), *t*(104,411) = 9.85, *p* <.001, the difference was negligible, Cohen’s *d* = 0.07, [95% CI = 0.05, 0.08].

### Loss Limits in 2021

In 2021, 9.1% of Norsk Rikstoto customers changed their loss limit at least once. Among these, the average number of changes was 1.32 during the year (SD = 1.27, range = 1–29), with a median of 1 (Q25 = 1, Q75 = 1). In total, 21,574 new loss limits were set, with an average amount of 12,845.45 NOK (SD = 21,751.64, range = 0.5–1,500,000). The median size of newly set limits was 10,000 NOK (Q25 = 5,000, Q75 = 20,000).

### The Relationship between Loss Limit in 2021 and Gambling Behavior in 2023

Table 1 shows the relationship between loss limits set in 2021 and gambling expenditure in 2023. Of the customers, 15.1% did not set a personal loss limit, while 67.1% set limits between 0.5 and 5,000 NOK. Overall, higher loss limits were associated with higher gambling expenditures. Customers in the 0.5-5,000 NOK category had the lowest mean and median expenditure (7,885 NOK and 1,299 NOK, respectively), followed by those who did not set a limit (12,320 NOK and 2,303 NOK). Customers with loss limits between 12,001 and 20,000 NOK had the highest mean and median expenditures (153,602 NOK and 60,450 NOK). The standard deviation in this group (498,526 NOK) was more than twice that observed in each of the other categories. Expenditures remained elevated across all categories above 5,000 NOK.

**Table 1:** The relationship between loss limit 2021 and expenditure in 2023

The relationship between loss limit 2021 and expenditure in 2023								
	Expenditure (in NOK)							
Loss limit	*N*	Min	Max	Mean	SD	Median	Q25	Q75
Did not set loss limit	15,722	2	673,282	12,320	32,973	2,303	528	9,768
0.5–5,000	70,010	0.2	2,313,267	7,885	29,229	1,299	368	5,512
5,001–12,000	6,941	8	7,499,400	52,361	124,010	21,297	4,101	63,332
12,001–20,000	11,478	2	31,761 394	153,602	498,526	60,450	10,757	181,484
20,001–50,000	144	93	1,617,835	121,370	204,007	52,831	13,823	146,682
50,001 or more	118	20	1,513,026	126,386	207,724	41,999	8,872	172,729
Total	104,413	0.2	31,761 394	27,818	176,882	2,240	480	12,069

Note

**Fig. 2:**
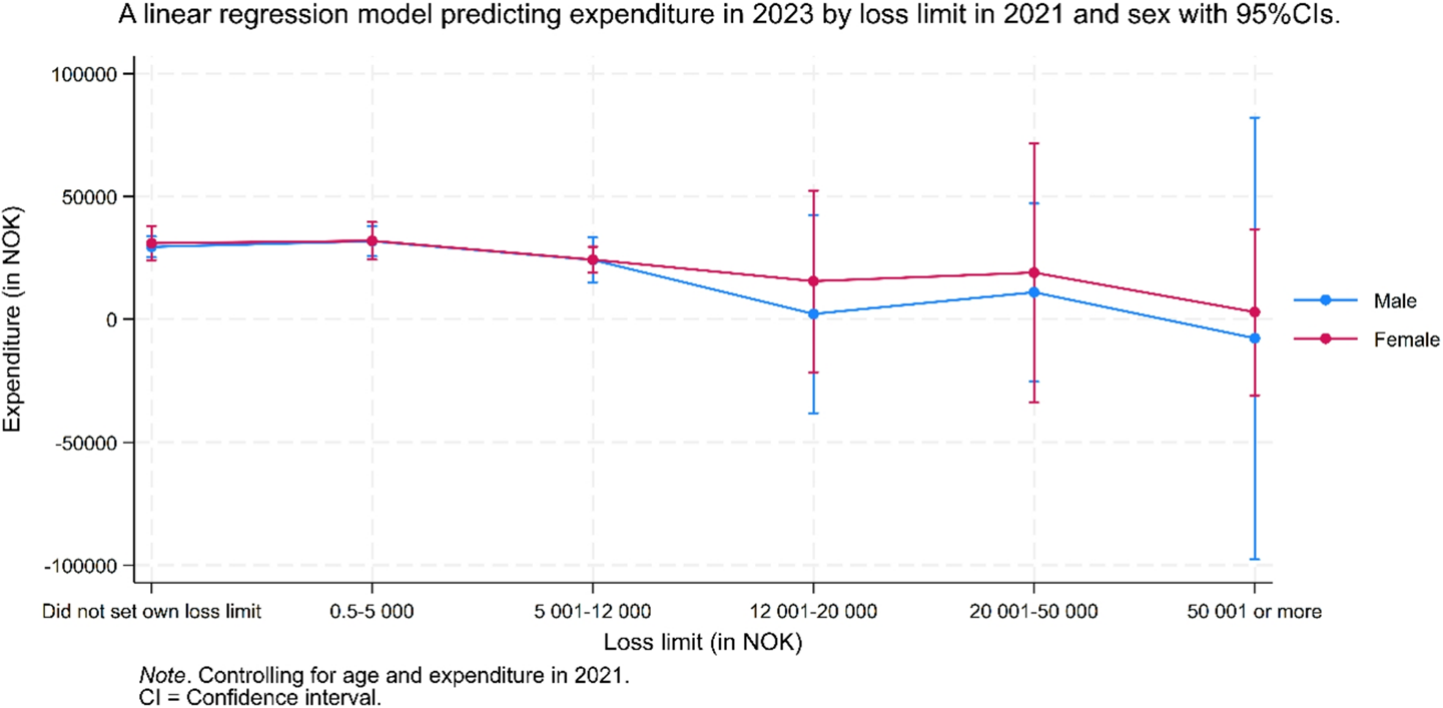
A linear regression model predicting expenditures in 2023 by loss limit in 2021

Figure 2 shows the predicted expenditure in 2023 based on loss limits set in 2021, stratified by gender. No clear association was observed between 2021 loss limit categories and expenditure in 2023 for either men or women. Uncertainty was greater at higher loss limit categories. The 95% confidence intervals in the highest loss limit categories encompassed the predicted expenditure estimates of all other categories, suggesting that meaningful differences could not be determined.

Table 2shows the relationship between loss limits set in 2021 and net winnings in 2023. Overall, higher loss limits were associated with lower net winnings. Customers in the 0.5-5,000 NOK category had the highest mean and median net winnings (i.e., the lowest average losses) (-3,063 NOK and -746 NOK), followed by those who did not set a loss limit (-4,447 NOK and -1,179 NOK). Net winnings were substantially lower among customers with higher loss limits. Mean losses peaked in the 12,001-20,000 NOK loss limit category (-40,173 NOK), while the largest median losses were observed in the 20,001-50,000 NOK category (-22,061 NOK). The standard deviation in the 12,001-20,000 NOK group (192,840 NOK) was markedly higher than in all other categories. Net winnings remained low across all categories above 5,000 NOK.

**Table 2:** The relationship between loss limit 2021 and net winnings in 2023

The relationship between loss limit 2021 and net winnings in 2023								
	Net winnings (in NOK)							
Loss limit	*N*	Min	Max	Mean	SD	Median	Q25	Q75
Did not set loss limit	15,722	−257,277	932,548	−4,447	17,590	−1,179	−4,606	−288
0.5–5,000	70,010	−305,433	643,257	−3,063	11,653	−746	−2,880	−192
5,001–12,000	6,941	−271,858	1,585,665	−17,338	43,271	−8,578	−25,565	−1,473
12,001–20,000	11,478	−1,651,843	11,015,454	−40,173	192,840	−21,167	−63,804	−3,450
20,001–50,000	144	−226,511	257,482	−37,255	54,515	−22,061	−57,537	−4,892
50,001 or more	118	−281,755	287,719	−39,140	76,115	−17,570	−57,470	−2,041
Total	104,413	−1 651,843	11,015,454	−8,387	67,084	−1,137	−5,544	−263

Note

**Fig. 3:**
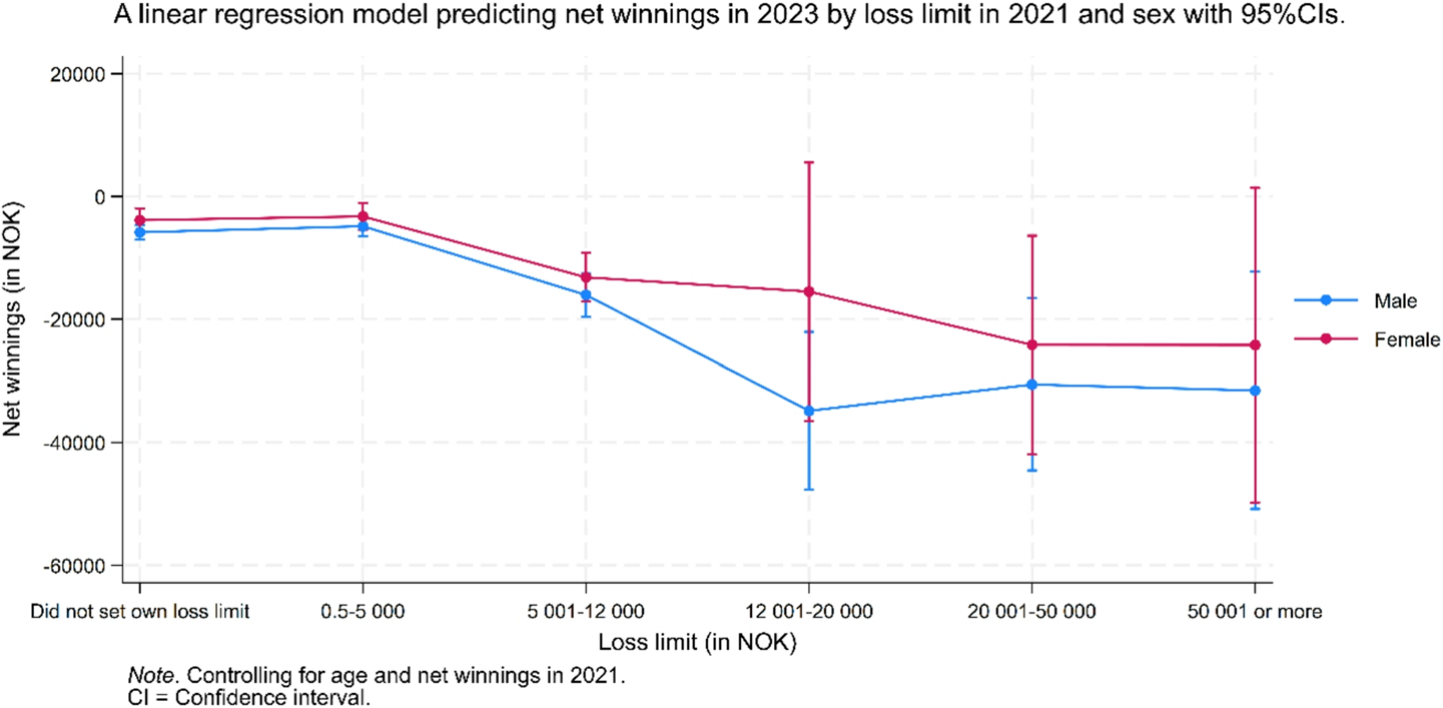
A linear regression model predicting net winnings in 2023 by loss limit in 2021

Figure 3 shows the predicted net winnings in 2023 based on loss limits set in 2021, stratified by gender. Overall, predicted net winnings tended to be lower (i.e., larger losses) in the three highest loss limit categories compared to the three lowest. Among men, net winnings were lowest in the 12,001–20,000 NOK loss limit category, whereas among women, they were lowest in the 20,001–50,000 NOK category. Men incurred higher losses than women across all loss limit categories; however, the wide 95% confidence intervals in the two highest loss limit categories, particularly among women, indicate substantial overlap in net winnings between genders. Additionally, the wide confidence interval in the highest loss limit category also encompassed the predicted net winnings estimates of all other categories, suggesting that no clear differences could be determined.

Table 3 shows the relationship between loss limits set in 2021 and the number of bets placed in 2023. Overall, higher loss limits were associated with a greater number of bets placed. Customers in the 0.5-5,000 NOK category placed the fewest bets on average (84 bets;median: 10), followed by those who did not set a limit (140 bets; median: 18). The highest mean number of bets was observed in the 12,001-20,000 NOK category (934 bets), while the highest median was found in the 20,001-50,000 NOK group (390 bets). The standard deviation in the 50,001 NOK or more group was the largest observed (2,365 bets). The number of bets remained elevated across all categories above 5,000 NOK.

**Table 3:** The relationship between loss limit 2021 and bets placed in 2023

The relationship between loss limit 2021 and bets placed in 2023								
	Number of bets placed							
Loss limit	*N*	Min	Max	Mean	SD	Median	Q25	Q75
Did not set loss limit	15,722	1	16,272	140	522	18	4	77
0.5–5,000	70,010	1	32,993	84	386	10	3	45
5,001–12,000	6,941	1	31,391	503	1,180	134	25	522
12,001–20,000	11,478	1	63,738	934	2,009	315	52	1,047
20,001–50,000	144	1	12,363	896	1,484	390	63	1,063
50,001 or more	118	1	16,418	908	2,365	277	52	694
Total	104,413	1	63,738	216	873	17	4	88

Note

**Fig. 4:**
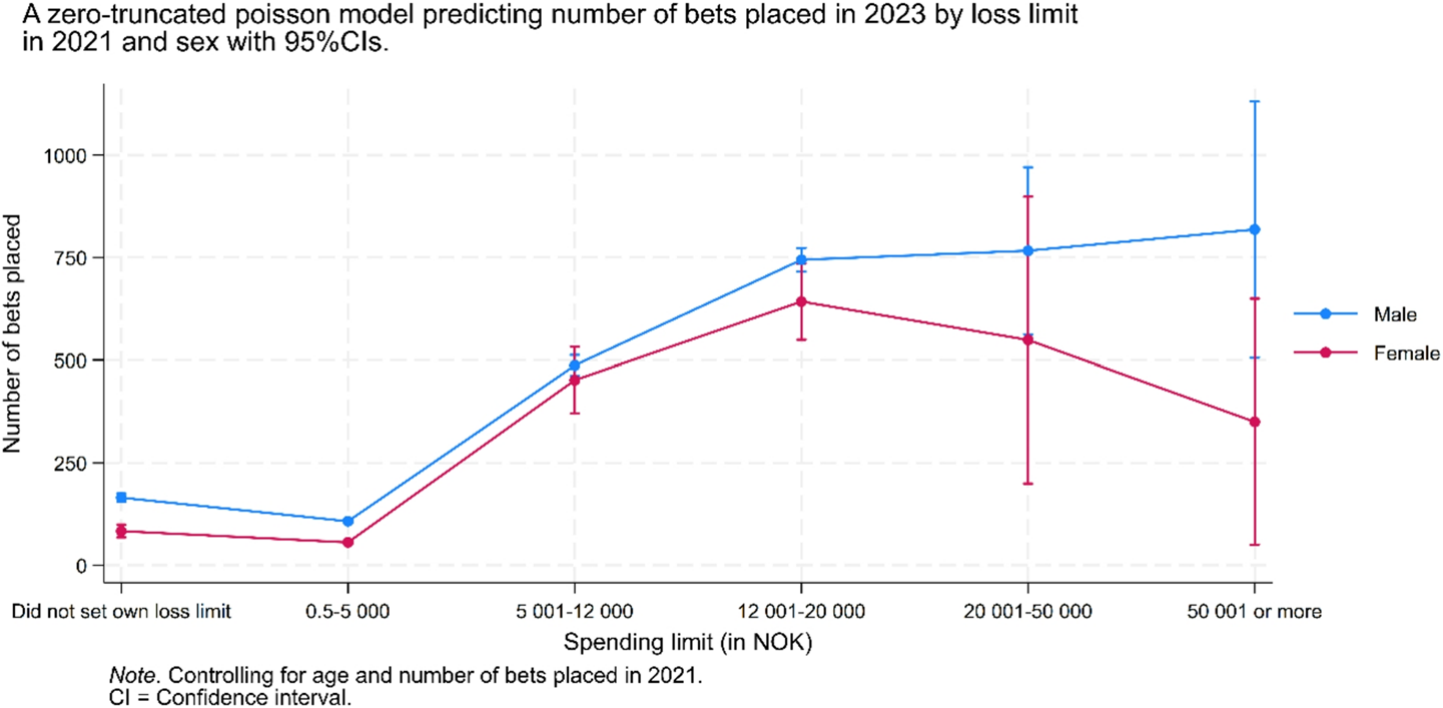
A zero-truncated poisson model predicting number of bets placed in 2023 by loss limit in 2021

Figure 4 shows the predicted number of bets in 2023 based on 2021 loss limits, stratified by gender. Overall, the predicted number of bets tended to be lower in the three lowest loss limit categories compared to the three highest. Among men, the number of bets peaked in both the 12,001–20,000 NOK and 50,001 NOK or more categories, whereas among women, the peak occurred in the 12,001–20,000 NOK category. Men placed more bets than women across all loss limit categories; however, the wide 95% confidence intervals in the 5,001–12,000 NOK, 20,001–50,000 NOK, and 50,001 NOK or more categories – particularly among women – indicate substantial overlap in betting activity between genders. Additionally, the wide confidence interval in the highest loss limit category overlapped with the predicted estimates of all other categories, suggesting that no clear differences across the categories could be determined.

Table 4 shows the relationship between loss limits set in 2021 and the risk of PrG in 2023. Overall, higher loss limits were associated with a greater likelihood of being classified as at moderate-to-high risk of PrG. Customers in the 0.5-5,000 NOK loss limit category had the lowest proportion of individuals at moderate-to-high risk of PrG (2.0%), followed by those who did not set a loss limit (4.4%). The highest proportion was observed in the 12,001-20,000 NOK group (46.0%). The proportion of individuals at moderate-to-high risk of PrGremained elevated across all categories above 5,000 NOK.

**Table 4:** The relationship between loss limit in 2021 and risk of gambling problems in 2023

The relationship between loss limit in 2021 and risk of gambling problems in 2023			
Loss limit	*N*	No-to-low	Moderate-to-high
Did not set loss limit	15,722	95.6%	4.4%
0.5–5,000	70,010	98.0%	2.0%
5,001–12,000	6,941	79.8%	20.2%
12,001–20,000	11,478	54.0%	46.0%
20,001–50,000	144	58.3%	41.7%
50,001 or more	118	58.5%	41.5%

Note. No-to-low = no-to-low risk of gambling problems; Moderate-to-high = Moderate to high risk of gambling problems

**Fig. 5:**
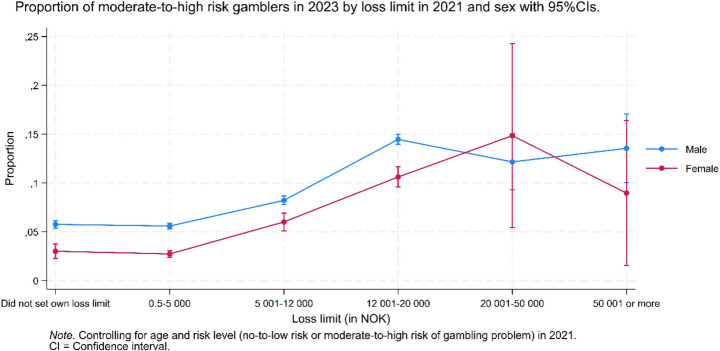
A binary logistic regression model to analyze the proportion of moderate-to-high risk gambling in 2023 by loss limit in 2021

Figure 5 shows the predicted proportion of moderate-to-high risk gamblers in 2023 based on loss limits set in 2021, stratified by gender. Overall, the predicted proportion of moderate-to-high risk gamblers was lower in the three lowest loss limit categories compared to the three highest. Among men, the highest predicted proportion was observed in the 12,001–20,000 NOK category, whereas among women, the highest was found in the 20,001–50,000 NOK category. Men had a higher predicted proportion of moderate-to-high risk gamblers than women across all loss limit categories; however, the wide 95% confidence intervals in the two highest categories, particularly among women, indicate substantial uncertainty and overlap between genders. Additionally, the wide confidence intervals in the two highest loss limit categories overlapped with several other categories, put restrictions on the ability to conclude about differences across groups.

**Fig. 6:**
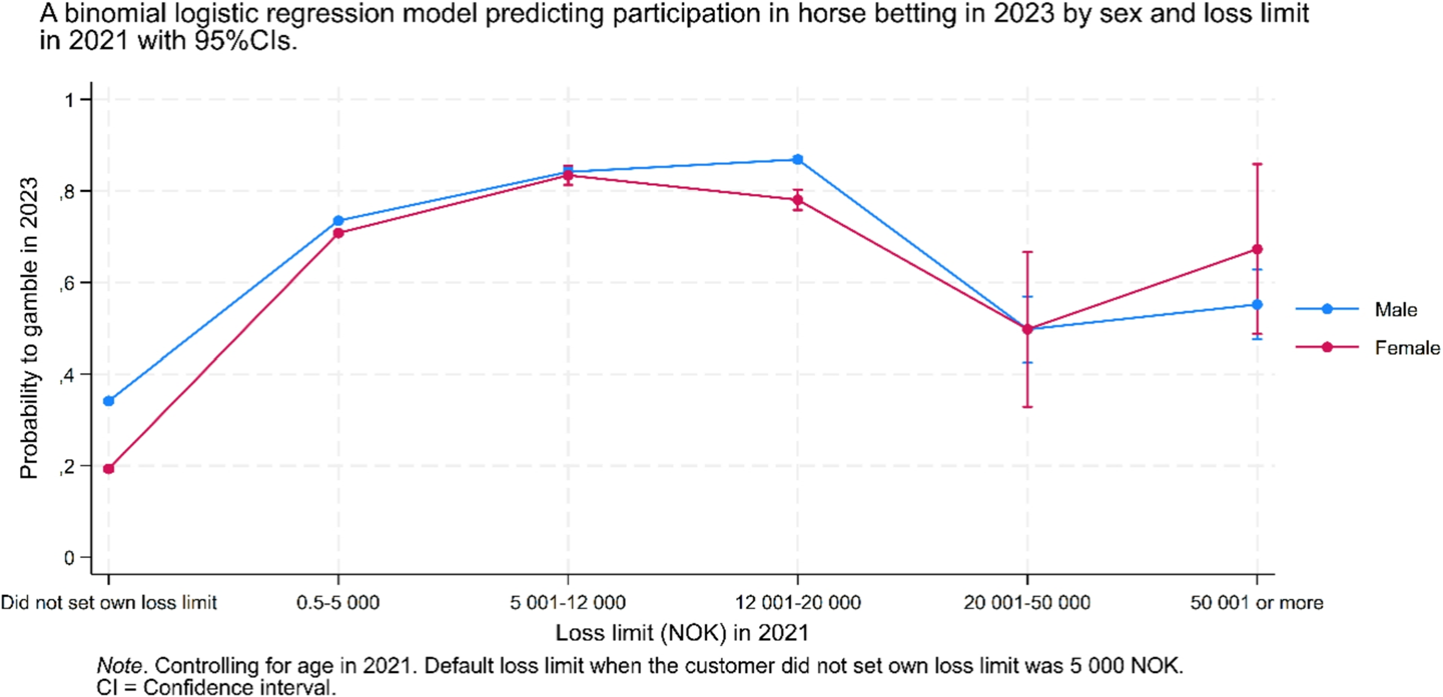
A binomial logistic regression model predicting participation in horse betting in 2023 by sex and loss limit in 2021

Figure 6 shows the predicted probability of gambling in 2023 based on loss limits set in 2021, stratified by gender. The results indicate a curvilinear association, with the probability peaking among men in the 20,001–50,000 NOK loss limit category and among women in the 5,001–12,000 NOK category. In the highest loss limit group, the probability increased slightly for women but remained stable for men compared to the preceding loss limit category.

## Discussion

The present study investigated whether the size of loss limits set in 2021 could predict gambling behavior and risk of moderate-to-high risk gambling in 2023. Across most outcomes (i.e., net winnings, number of bets placed, and risk classification), higher loss limits were associated with more intensive gambling behavior. The highest levels of gambling activity were observed up to the 12,001–20,000 NOK loss limit category, beyond which gambling behavior remained elevated but exhibited greater variability. This pattern was particularly evident among men, whose gambling behavior intensified more predictably and steeply across loss limit categories. These findings are consistent with previous research showing that pre-commitment tools such as loss limits are associated with reductions in gambling intensity and frequency (Delfabbro & King, 2021), yet they also extend our understanding by demonstrating long-term predictive effects over a two-year period.

Although expenditure did not show a clear association with higher loss limits, the negative association with net winnings (i.e., higher loss limits predicting greater losses) suggests that players, especially men, in higher loss limit categories were more likely to lose more money than they won. While some individuals may possess the financial means or self-regulation to withstand substantial gambling losses without harm (Langeland et al., 2022), this is unlikely to be the case for all. For many, elevated expenditures on gambling may thus contribute to financial distress, strained relationships, and a range of emotional or psychological consequences (Langham et al., 2016). The observed persistence of gambling at higher loss limits highlights their potential to sustain harmful involvement. These concerns are amplified by findings showing that individuals with lower income and education levels are more likely to be classified as moderate- or high-risk gamblers (Pallesen et al., 2023). As a result, individuals with limited financial resources may be especially susceptible to harm under higher loss limits.

Although being classified as a moderate- or high-risk gambler does not necessarily indicate a fully developed gambling problem, it signals problematic gambling tendencies based on the observed behavior (Rikstoto, 2022). Given that nearly half of gamblers with a loss limit in the 12,001–20,000 NOK range were classified as having moderate-to-high risk of developing PrG, and that this proportion remained elevated across all higher limit categories, raise concern about the risks associated with higher loss limits. Loss limits that are not based on financial capacity may unintentionally promote harmful spending among those least able to absorb the consequences. In this context, implementing loss limits tailored to income may offer a more equitable and protective approach to harm minimization (Langeland et al., 2022). Another complementary strategy includes targeted interventions, such as proactive phone calls to individuals with higher personal loss limits, which have shown promise in reducing gambling-related harm (Jonsson et al., 2023). However, future studies should further examine how socioeconomic factors, such as income, debt, and financial literacy, interact with gambling expenditure and loss limit behavior, in order to inform more targeted and effective regulation.

While higher loss limits were associated with elevated risk, the opposite trend was observed among those with lower limits. Among customers with loss limits in the 0.5–5.5,000 NOK range, only 2.0% were classified as moderate-to-high risk gamblers. This suggests that a lower upper loss limit cap may offer long-term protection, helping to reduce the population-level prevalence of problem gambling. A mandated cap of 5,000 NOK per month could thus serve as a valuable public health measure. Alternatively, if such a cap is not implemented, control strategies such as warning messages, encouragement to lower self-imposed limits, or exclusion from marketing, could help mitigate risks among individuals with higher loss limits. While protecting vulnerable individuals is essential, it is also important to balance consumer protection with personal autonomy, as overly restrictive limits may lead to unintended consequences such as migration to unregulated gambling markets. However, a 2018 study on Norwegian gamblers found that even when players had the option to switch to other gambling sites after reaching their loss limit, the vast majority chose not to, including those classified as high-risk gamblers (Auer et al., 2018). This suggests that loss limits effectively curb further gambling for most individuals, including those most at risk. Still, it remains unclear whether these effects would persist if caps were made more stringent, highlighting the need for continued research.

Gender differences were also observed, with men setting higher loss limits and displaying more intense gambling behaviors, possibly due to greater impulsivity and risk-seeking tendencies (González-Ortega, 2013). Such traits and tendencies may lead men to gamble more frequently and intensely under high loss limits, making them more prone to developing gambling problems. Interestingly, women with the same loss limits as men tended to gamble more cautiously. In other words, while men generally set higher limits, even within the same loss limit category, especially the highest ones, men gambled more intensely. The cautious gambling approach observed among women may stem from socioeconomic disparities. With generally lower earnings and higher economic vulnerability (Statistics Norway), women may prioritize financial stability, viewing gambling losses as a more substantial risk to their financial well-being.

Although women generally set lower loss limits and overall are engaged in more cautious gambling behavior compared to men, they still exhibited the same trends as their gambling intensity increased with higher loss limits. The wide 95% confidence intervals observed among women with higher loss limits may be explained by their lower representation in the investigated population compared to men. However, the results may also indicate that while most women tend to gamble less intensely, there is a subset whose gambling intensity matches or even exceeds that of most men. Given the telescoping effect (Syvertsen et al., 2023b), early intervention for these women could be crucial to prevent the rapid progression of gambling-related problems. Further research on female gamblers is still warranted to better capture the nuances of their gambling behaviors and risk patterns.

### Strengths and Limitations of the Present Study

The present study benefits from using a full population dataset rather than relying on a sample, which generally minimizes sampling error and increases the accuracy and generalizability of findings (Thygesen & Ersbøll, 2014). Since the whole population was studied, testing for statistical significance was deemed redundant; however, although it in some cases still may be valuable, as it can reveal underlying patterns and enhance the generalizability of findings to other populations (Alexander, 2015). Additionally, account-based gambling data offers objective, real-time insights into gambling behavior, avoiding biases commonly associated with self-reported data. The present study offers a rare longitudinal perspective in gambling research as loss limits were investigated as a predictor of objectively recorded gambling behavior two years later. Still, some limitations should be noted. The loss rollover system, which allows loss limits to exceed 20,000 NOK, complicated the interpretation, as players with rollovers may behave differently. High standard deviation and wide confidence intervals in several metrics indicate significant variability in behavior, suggesting that loss limit associations vary widely and may obscure key subgroup differences, potentially limiting the impact of responsible gambling recommendations. The 2023 risk classification was derived from an operator algorithm that includes changes in loss limits, meaning this outcome is not entirely independent of our main predictor. Moreover, since the present study does not account for whether individuals reached their loss limits, uncertainty arises regarding the ability of loss limits curbing gambling behavior. Even though the dataset explores a diverse set of variables, the analysis overlooks important factors like socioeconomic status, mental health, and behavioral traits, which have been shown to be strongly gambling behavior (Allami et al., 2021; Pallesen et al., 2023; Myrseth et al., 2009). Further, loss limit categorization was based on the last limit set in 2021, which not necessarily reflect the overall approach to loss limits by the customers. In addition, those who did not place bets in 2023 were removed from the analyses. We cannot rule out that these factors to some extent can limit the validity of the findings. The customers may increase their losses any time, however it will take two days for such change to be effected. Reductions in loss limits will, however, be effected immediately.

### Implications for Practice and Future Research

The findings from the present study indicate that loss limits could be a valuable tool for identifying potential at-risk gamblers or at least those with intensive gambling behavior. Implementing stricter loss limit caps could reduce harmful behaviors by preventing excessive losses and lowering the risks associated with increased gambling intensity. While the present study focused on horse race betting, further research should assess how well loss limits predict gambling patterns across a wider range of gambling products and whether they remain reliable predictors beyond two years. Additionally, examining variations within risk categories as well as individual differences would provide deeper insights into gambling behavior, enabling more targeted, personalized interventions that cater to the unique risk profiles of different gamblers. Data on account closures and self-exclusions were not available in the present study, hence future research should examine whether loss limits predict such behavioral outcomes as they may provide additional insights into gambling-related risk.

## Conclusion

The present study demonstrated that loss limits set in 2021 could, at least at the group level, predict gambling behavior in 2023. Higher loss limits were associated with increased gambling activity and a higher likelihood of being classified as a moderate-to-high risk gambler. The findings reveal significant gender differences, with men engaging in more intense gambling across all loss limits. These results underscore the need for regulatory measures to mitigate gambling risks, while further research is needed to fully understand long-term gambling patterns and individual differences within various loss limit groups.

## Supplementary information

Below is the link to the electronic supplementary material.

Supplementary file1 (TXT 31.5 KB)
